# Pathogenic mechanisms of intestinal pneumatosis and portal venous gas: should patients with these conditions be operated immediately?

**DOI:** 10.1186/s40792-015-0104-7

**Published:** 2015-10-15

**Authors:** Akira Mitsuyoshi, Shinshichi Hamada, Tsuyoshi Tachibana, Teppei Momono, Hiroki Aoyama, Yuhei Kondo, Kenta Inoguchi, Daiju Yokoyama, Masayuki Nakau, Sato Suzaki, Hiroshi Okabe, Ken Yanagibashi

**Affiliations:** Department of Surgery, Otsu Municipal Hospital, 2-9-9 Motomiya, Otsu, Shiga 520-0804 Japan; Department of Pathology, Otsu Municipal Hospital, Otsu, Japan

## Abstract

We aimed to histologically observe portal venous gas (PVG)-causing intestinal pneumatosis (IP) and evaluate pathogenic mechanisms and therapeutic strategies, including decisions on whether emergency surgery should be performed. Autopsy was performed in two cases of nonocclusive mesenteric ischemia (NOMI). We directly histologically observed the pathogenic mechanisms of IP caused by gas-producing bacteria and IP considered to be caused by mechanical damage to the intestinal mucosa. IP can be classified hypothetically into the following types according to pathogenesis: (1) infection, (2) rupture (damage) of the intestinal mucosa + increased intestinal intraluminal pressure, and (3) mixed type. In cases of IP caused by gas-producing bacteria or IP associated with intestinal wall damage extending beyond the mucosa to the deep muscular layer, emergency surgery should be considered. However, it is highly possible that patients who test negative for infection with gas-producing bacteria whose intestinal wall damage remains only in the mucosa can be conservatively treated.

## Background

For a long time, portal venous gas (PVG) has been considered to be a sign of poor prognosis in abdominal diseases [1, 2]. Especially when PVG is caused by intestinal necrosis due to ischemia, the mortality rate is high even after emergency laparotomy with intestinal resection [1, 3]. Although PVG-causing intestinal pneumatosis (IP) can be broadly classified into IP caused by intestinal wall infection with gas-producing bacteria and IP caused by mechanical rupture of the intestinal mucosal wall [4, 5], no report has clearly described the actual processes of the conditions by using histological findings. In the two cases of nonocclusive mesenteric ischemia (NOMI) that we experienced, we directly observed IP from a histopathological perspective. We report a summary of the pathogenic mechanisms and primary diseases, as well as therapeutic strategies and prognosis, in these two cases, along with a brief literature review.

## Case presentation

### Case 1

**Fig. 1:**
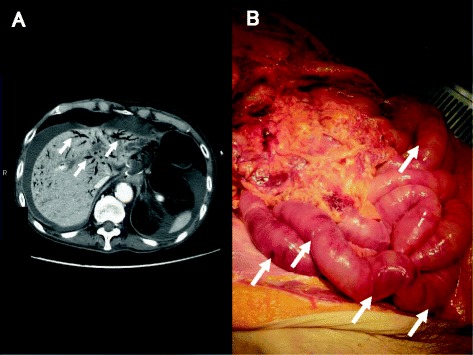
**a** CT for case 1. PVG spreads through the whole liver (*arrow*). **b** Autopsy photos for case 1. Segmental ischemic change was recognized at the wide range of the small intestine (*arrow*)

**Fig. 2:**
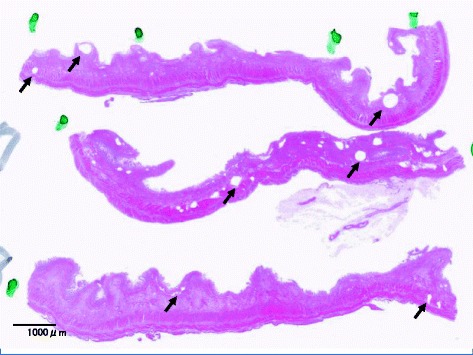
Cut surface of the small intestine for case 1. Lots of IP were found at all layers of the small intestine (*arrow*)

**Fig. 3:**
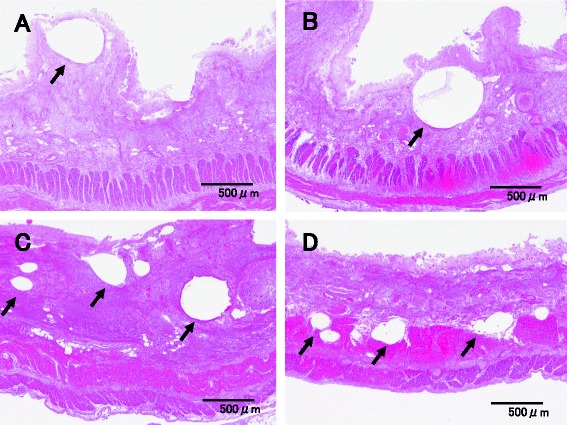
Microscopic findings of case 1 (HE). Intestinal gas might have gradually seeped into the ruptured mucosal surfaces (**a**, **b**), submucosa (**c**), and muscular layer (**d**) as IP (*arrow*)

**Fig. 4:**
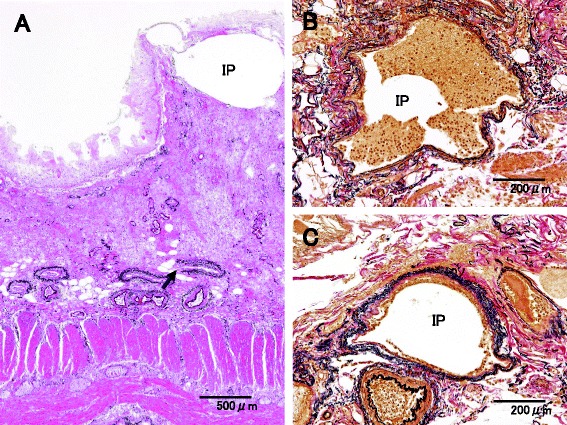
Microscopic findings of case 1. **a** IP and wall-damaged vessel (*arrow*) existed simultaneously at the submucosa that fell into necrotic change (Elastica van Gieson + HE). **b**, **c** IP that might have moved into the vessel from the submucosa (Elastica van Gieson)

**Fig. 5:**
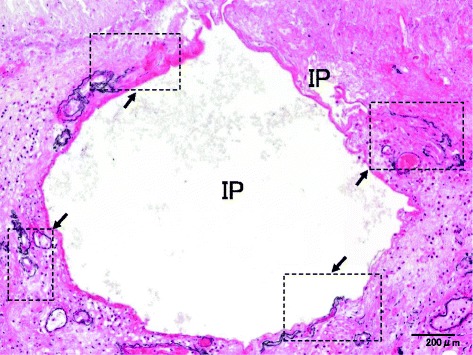
Microscopic findings of case 1(Elastica van Gieson + HE). At the submucosal layer that fell into necrosis, IP was surrounded by lots of wall-damaged vessels (*arrow*)

**Fig. 6:**
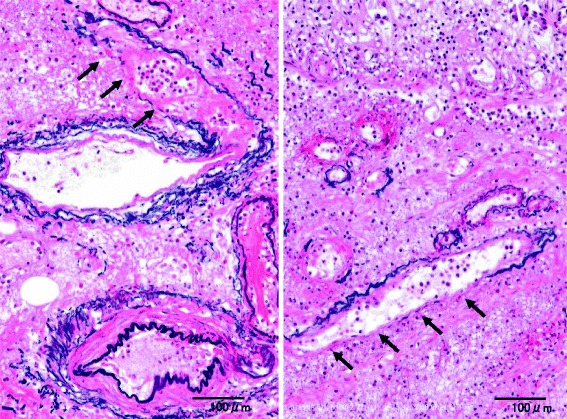
Microscopic findings of case 1(Elastica van Gieson + HE). The lack of elastic fiber and smooth muscle was observed in wall-damaged vessels (*arrow*) at the submucosal layer that fell into necrosis. That may be the entrance of IP

Case 1 is a 79-year-old man with hypertension and old myocardial infarction. Although total gastrectomy and lymphadenectomy were performed for gastric cancer, dehydration triggered the onset of NOMI after the surgery. Because the NOMI itself was mild, conservative therapy was initiated at first. However, heart failure concomitantly occurred, and he died 4 days after surgery. Since the onset of NOMI, the patient had presented with few signs of infection, and the main clinical symptoms had been impaired intestinal peristalsis due to intestinal ischemia and abdominal distension (Fig. 1). According to autopsy results, no parenchymal obstructive lesion was observed in the major arteries of the superior mesenteric artery (SMA) and inferior mesenteric artery (IMA), as well as vascular insufficiency in the mesentery. Histopathological examination revealed necrosis and shedding of the small intestinal mucosa and venous congestion in the submucosal or muscular layer. Moreover, we observed lots of IP at all layers of the small intestine (Figs. 2 and 3). IP and wall-damaged vessel exist simultaneously at the submucosa that fell into necrotic change, some IP might have moved into the vessel from the submucosa, and others were surrounded by lots of wall-damaged vessels (Figs. 4, 5, and 6). No gas-producing bacterium was detected in the intestinal wall. The necrosis of mucosal surfaces was assumed to have concomitantly occurred with the onset of NOMI. We assumed that intestinal gas migrated from the ruptured mucosal surfaces into the intestinal wall consequently because of the high intraluminal pressure, and then IP occurred. The direct cause of death was determined as heart failure that was not directly associated with the NOMI.

### Case 2

**Fig. 7:**
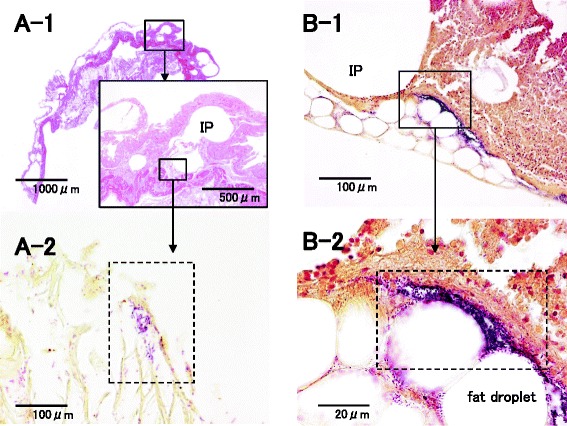
Cut surface and microscopic findings of the small intestine for case 2. **a**-**1**, **2** Lots of IP were found at all layers, and some colonies of *Clostridium perfringens* (Welchii) were formed around IP (**a**-**1** HE, **a**-**2** Gram). **b**-**1**, **2** Other colonies of *Clostridium perfringens* (Welchii) were formed in areas slightly away from IP (Gram)

**Fig. 8:**
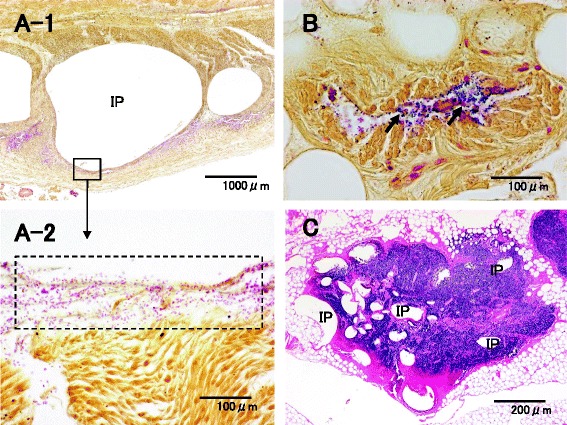
Microscopic findings of the small intestine for case 2. **a**-**1**, **2** Lots of colonies of *Escherichia coli* were formed around IP (Gram). **b** IP and colonies of *Clostridium perfringens* (Welchii) were observed in the blood vessels. (Gram). **c** IP of *Clostridium perfringens* (Welchii) was observed in lymph nodes in the intestinal wall (HE)

Case 2 is an 82-year-old man. After endoscopic submucosal dissection (ESD) for early gastric cancer, repetitive bleeding occurred. Although blood pressure was relatively low 5 days after ESD, high temperature was not recognized. Fever and acute low blood pressure happened 6 days after ESD; he rapidly went into shock the next day. Emergency laparotomy for hemostasis was considered but could not be performed because his general condition rapidly deteriorated. He developed NOMI and sepsis, and then he died 7 days after ESD. Although autopsy did not reveal vascular insufficiency in the mesentery, full-thickness necrosis was observed in a wide area ranging from the stomach to the small intestine. Histopathological examination revealed IP in many parts of the intestinal wall and confirmed that gas-producing bacteria such as *Clostridium perfringens*, *Escherichia coli*, and *Klebsiella pneumoniae* had formed colonies near the lesions (Figs. 7 and 8). Furthermore, pneumatosis was also observed in the blood vessels and lymph nodes in the intestinal wall. Gram-negative bacilli such as *E. coli* and *K. pneumoniae* showed a strong tendency to form colonies around the pneumatosis, whereas *C. perfringens* (Welchii), a Gram-positive bacillus, was found likely to form colonies in areas slightly away from pneumatosis. In this case, we assumed that the shock associated with the bleeding after ESD was complicated by the severe infection and NOMI and that the gas directly produced in the wall by the infecting gas-producing bacteria caused the IP.

### Discussion

PVG was first reported in neonatal cases by Wolfe et al. in 1955 [4], while the first adult cases were reported by Susman et al. in 1960 [5]. Since then, PVG and IP have often been reported in cases of severe necrotizing enterocolitis associated with ischemia, such as SMA embolism and NOMI. In the past, PVG and IP were regarded as signs of poor prognosis [1, 2] and pathological conditions for which emergency surgery such as intestinal resection should first be considered. In recent years, with wide adoption of multidetector-row computed tomography (MDCT), the number of cases diagnosed in the relatively early stage or cases without severe infection has also increased [6], and a number of conservatively treated cases have also been reported [7–9]. However, in our literature search, we did not find any report of a detailed morphological analysis of the actual mechanisms; thereby, IP was caused by infection, without infection, or by mechanical mucosal damage.

In the two cases of NOMI that we recently experienced, we made the hypothesis about the pathogenic mechanisms of IP from a histological perspective. In cases of not only NOMI but also intestinal ischemia, damage due to these conditions first occurs in the mucosa and then gradually spreads to all muscular layers. However, in case 1, the mucosal necrosis and consequent mucosal rupture (laceration) were mild, and no damage caused by the ischemia was observed in the submucosal layer, muscular layer, or serosa. Intestinal gas was observed that might have migrated to the lacerated mucosa and seeped into the deep muscular layer and then further into wall-damaged blood vessels; no bacterial infection was observed in the intestinal wall. Unfortunately, the patient in this case died of heart failure. However, we assume that the NOMI in this case could have been treated conservatively without performing surgery.

In case 2, although the autopsy findings were consistent with findings in NOMI, the findings in intestinal infection associated with necrosis were histologically more apparent than those in necrosis caused by intestinal vascular insufficiency. We observed that gas was directly produced in the intestinal wall and migrated into blood vessels. This pathological condition would ultimately lead to perforation and was normally indicated for emergency surgery aimed at intestinal resection. However, the patient rapidly went into shock, because of which we missed the timing of surgery. This case characteristically showed that a Gram-negative bacillus formed colonies around the pneumatosis, while a Gram-positive bacillus tended to form colonies in areas slightly away from the pneumatosis. In order to determine whether these findings are universal trends, future research reports are needed.

**Fig. 9:**
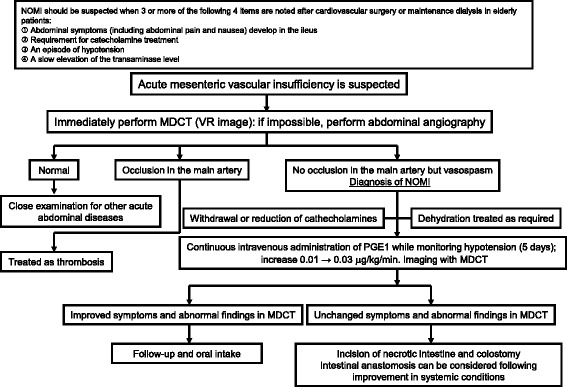
NOMI diagnosis and treatment in our hospital [13]

NOMI that is not associated with parenchymal occlusion of major arteries is a disease with a high mortality rate because the start of treatment is often delayed because of the scarcity of characteristic symptoms and difficulty in establishing the diagnosis. Although the characteristic finding of spasm of the major arteries, which was reported by Boley [10] and Siegelman et al. [11], has been used as basis for the angiographic diagnosis of NOMI, only a few reports described this finding as actually helpful for early diagnosis of NOMI. The first case was reported by Ende in 1958, and discussion on the diagnostic criteria based on angiographic findings was started around 1974 [12]. In those days, computed tomography was not widely adopted yet and NOMI could be diagnosed only after laparotomy or autopsy revealed extensive necrosis or perforation in all the layers of the intestinal tract. In severe, advanced NOMI, the area of intestinal necrosis is extensive; PVG often coexists, and the mortality rate is high. In 2007, we presented that MDCT is a useful alternative to angiography for the early diagnosis of NOMI [13]. Since then, the disease concept of NOMI has gradually been recognized in wide areas. Moreover, as the use of MDCT has rapidly expanded, reports of cases in which NOMI was diagnosed and treated early or those in which patients even with NOMI associated with PVG were conservatively treated and survived have been increasing. We apply adequate fluid replacement and bolus-continuous intravenous injection of prostaglandin E1 as the first choice for the treatment of early NOMI (Fig. 9).

Based on the results of this pathological observation, we made the hypothesis about classification and summarized the pathogenic factors of PVG-causing IP into (1) infection, (2) mucosal rupture + increased intraluminal pressure, and (3) mixed type (Table 1). When infection is the main cause, gas-producing bacteria such as *C. perfringens*, *E. coli*, and *K. pneumoniae* infect and colonize the intestinal wall. Although the condition is often transited from (2) or (3), which is described below, mucosal rupture (damage) is not always involved. In such a case, patients are highly likely to have severe infection or to have already developed sepsis, and a fatal outcome is likely. When necrosis of the intestinal wall extends beyond the mucosa, the wall is easily perforated and infection progresses to diffuse peritonitis. Thus, emergency surgery aimed at removing infected lesions and preventing perforation should first be considered. This condition corresponds to sepsis caused by acute mesenteric artery thrombosis, severe NOMI as seen in case 2, and so on. When mucosal rupture that is mechanically caused by, for example, mucosal necrosis due to ischemia or trauma, is combined with increased intestinal intraluminal pressure, intestinal gas seeps into the intestinal wall and gradually migrates to the deep layer and further to blood vessels. In this case, infection does not always occur concomitantly, and the risk of perforation is also low when the extent of mucosal rupture is limited. Thus, emergency surgery is not always required. Mild NOMI, as seen in case 1, fits into this condition. In addition, the condition may occur in inflammatory bowel disease, gastric cancer, gastric ulcer, ileus due to various causes, severe constipation, blunt abdominal trauma, and so on. The mixed type consists of infection and mucosal rupture. Even in cases presenting with only mucosal rupture at first, if the rupture persists for a long period of time, it will be complicated by bacterial infection from the mucosa and progress to the above-described pathogenic factor (1).

**Table 1:** Our hypothesis about classification of IP

① Infection	Mechanism
	Gas-producing bacteria infect and colonize the intestinal wall. Mucosal rupture is not always involved.
	Clinical cases
	Sepsis caused by acute mesenteric ischemia and others.
	Prognosis
	Highly likely to have severe infection or to have already developed sepsis, and a fatal outcome is likely.
	Therapeutic strategies
	Emergency surgery should first be considered.
② Mucosal rupture + increased intraluminal pressure	Mechanism
	Intestinal gas seeps into the damaged mucosa and gradually migrates to the deep layer; infection does not always occur concomitantly.
	Clinical cases
	Inflammatory bowel disease, gastric cancer or ulcer, ileus, severe constipation, blunt abdominal trauma, and so on.
	Prognosis
	The risk of perforation is low when the extent of mucosal rupture is limited. If the rupture persists for a long time, it will be complicated by bacterial infection and progress to the above “infection.”
	Therapeutic strategies
	Emergency surgery is not always required.
③ Mixed type	① + ②

IP can be classified into three types according to pathogenesis: (1) infection, (2) rupture (damage) of the intestinal mucosa + increased intestinal intraluminal pressure, and (3) mixed type

## Conclusions

Regarding NOMI associated with IP, we have histopathologically made the hypothesis about the pathogenic mechanisms, summarized the diseases that can cause NOMI, and discussed therapeutic strategies and prognosis, along with a brief literature review.

In addition to NOMI are many other gastrointestinal diseases that cause PVG and IP. In gastrointestinal diseases associated with intestinal necrosis, the incidence rates of concomitant PVG and IP are high and fatal outcome is highly likely. Thus, whether PVG and IP are caused by infection or mechanical mucosal rupture should be definitively determined, and treatment should be applied based on pathological conditions. Even in patients with conditions complicated by PVG and IP, the chance of survival is good depending on the causative factors. Thus, treatment should not be easily abandoned.

## Consent

Written informed consent was obtained from the patient for publication of this case report and any accompanying images. A copy of the written consent is available for review by the Editor-in-Chief of this journal.

## References

[1] Liebman PR, Patten MT, Manny J, Benfield JR, Hechtman HB (1978). Hepatic-portal venous gas in adult: etiology, pathophysiology and clinical significance. Ann Surg.

[2] Hong JJ, Gadaleta D, Rossi P, Esquivel J, Davis JM (1997). Portal vein gas, a changing clinical entity. Report of 7 patients and review of the literature. Arch Surg.

[3] Bloom RA, Lebensart PD, Levy R, Craciun E, Anner H, Manny J (1990). Survival after ultrasonographic demonstration of portal venous gas due to mesenteric artery occlusion. Postgrad Med J.

[4] Wolfe JN, Evans WA (1995). Gas in the portal veins of the liver in infants; a roentgenographic demonstration with postmortem anatomical correlation. Am J Roentgenol Radium Ther Nucl Med.

[5] Susman N, Senturia HR (1960). Gas embolization of the portal venous system. Am J Roentgenol Radium Ther Nucl Med.

[6] Faberman RS, Maya-Smith WW (1997). Outcome of 17 patients with portal venous gas detected by CT. AJR.

[7] Kinoshita H, Shinozaki M, Tanimura H (2001). Clinical features and management of hepatic portal venous gas. Arch Surg.

[8] Muroya D, Hidaka A, Kojima S (2014). A case of hepatic portal venous gas and pneumatosis intestinalis caused by transit type ischemia. The Journal of The Kurume Medical Association.

[9] Atoji H, Sakai N, Tanji Y (2012). A patient of the acute phase with severe ischemic enteritis accompanied by portal venous gas who survived conservative treatment. Journal of Japanese Society of Gastroenterology.

[10] Boley SJ, Sprayregan S, Siegelman SS, Veith F (1977). Initial results from an aggressive roentgenological and surgical approach to acute mesenteric ischemia. Surgery.

[11] Siegelman SS, Sprayregen S, Boley SJ (1974). Angiographic diagnosis of mesenteric arterial vasoconstriction. Radiology.

[12] Ende N (1958). Infarction of the bowel in cardiac failure. N Eng J Med.

[13] Mitsuyoshi A, Obama K, Shinkura N, Ito T, Zaima M (2007). Survival in nonocclusive mesenteric ischemia. Early diagnosis by multidetector row computed tomography and early treatment with continuous intravenous high-dose prostaglandin El. Ann Surg.

